# A prospective observational study assessing the outcome of Sepsis in intensive care unit of a tertiary care hospital, Peshawar

**DOI:** 10.12669/pjms.323.9978

**Published:** 2016

**Authors:** Arslan Rahat Ullah, Arshad Hussain, Iftikhar Ali, Abdul Samad, Syed Tajammul Ali Shah, Muhammad Yousef, Tahir Mehmood Khan

**Affiliations:** 1 Dr. Arslan Rahat Ullah, FCPS. Department of Medicine & Allied, Northwest General Hospital & Research Centre, Peshawar, Khyber Pakhtunkhwa, Pakistan; 2 Dr. Arshad Hussain, MRCPI. Department of Medicine & Allied, Northwest General Hospital & Research Centre, Peshawar, Khyber Pakhtunkhwa, Pakistan; 3 Iftikhar Ali, Pharm.D, MPH. Department of Pharmacy, University of Swabi, Khyber Pakhtunkhwa, Pakistan. Department of Pharmacy Services, Northwest General Hospital & Research Centre, Peshawar, Khyber Pakhtunkhwa, Pakistan; 4 Prof. Abdul Samad, FRCP. Department of Medicine & Allied, Northwest General Hospital & Research Centre, Peshawar, Khyber Pakhtunkhwa, Pakistan; 5 Dr. Syed Tajammul Ali Shah, MBBS. Department of Medicine & Allied, Northwest General Hospital & Research Centre, Peshawar, Khyber Pakhtunkhwa, Pakistan; 6 Dr. Muhammad Yousef, MBBS. Department of Medicine & Allied, Northwest General Hospital & Research Centre, Peshawar, Khyber Pakhtunkhwa, Pakistan; 7 Dr. Tahir Mehmood Khan, PhD. School of Pharmacy, Monash University Malaysia, Bandar Sunway, Malaysia

**Keywords:** Sepsis, Septic shock, Pneumonia, Urosepsis, Infection

## Abstract

**Objective:**

The current study aims to explore the factors associated with outcome among patients with severe sepsis and septic shock admitted to the intensive care unit, Northwest General Hospital and Research Centre, Peshawar, Pakistan.

**Methods:**

A prospective observational study was carried out at intensive care unit of our hospital from February 2014 to October 2015. Data was collected using a structured format and statistical analysis was done using SPSS version 20®. Regression model was applied to identify the factors contributing to the outcome of severe sepsis and septic shock. P-value less than 0.05 was considered statistically significant.

**Results:**

Majority of the patients meeting the criteria of this study were male 147 (54.9%) with a mean age of 54.8. The most common source of sepsis was lung infections (42.2%) followed by urinary tract infections (18.7%), soft tissue infections (6.3%) abdominal infections (6%) and in 6.3% patients the source remained unknown. Further analysis has revealed that increase in number of days of hospitalization was observed to be slightly associated with the outcome of the treatment (1.086 [1.002 – 1.178], 0.046). Moreover, the risk of mortality was the higher among the patients with septic shock 22.161[10.055 – 48.840], and having respiratory, kidney and central nervous system complications. Overall it is seen that septic shock alone was found responsible to cause death among 32.0% of the patients (Model 1: R^2^ 0.32, p=0.000), and upon involvement of the organ complications the risk of mortality was observed to 42.0%.

**Conclusion:**

Chances of recovery were poor among the patients with septic shock. Moreover, those patients having respiratory and urinary tract infection are least likely to survive.

## INTRODUCTION

Sepsis is one of the most common, least-recognized illnesses in both the developed and developing world. It is the primary cause of death from infection despite advances in modern medicine, and carries long-term complications.^1^ Sepsis causes more deaths than prostate cancer, breast cancer and HIV/AIDS combined.^2^ It is estimated that more than 18 million people suffer from sepsis each year and more than 5 million of them die.^3^ Severe sepsis and septic shock are important causes of morbidity and mortality in patients admitted to intensive care units. These conditions are generally associated with multiple organ failure as final outcome.^4^

Sepsis occurs in approximately 2% of all hospitalizations in developed countries. Sepsis may occur in between 6 and 30% of all intensive care unit (ICU) patients However, there is substantial variation in the incidence of sepsis and severe sepsis due to the heterogeneity between ICUs. In general, more than 50% of severe sepsis patients will require intensive care services.^5^,^6^ It is evident that the mortality rate may have decreased in recent years but the incidence of severe sepsis and septic shock appears to be increasing, by 13.7% per year ultimately increasing overall deaths.^7^

The incidence of sepsis varies among different racial and ethnic groups.^8^ In the United States, the incidence of severe sepsis is estimated to be 300 cases per 100 000 population from the years 1999-2005. In the UK alone it claims between 36,000 to 64,000 lives per year.^4^ The mortality rate from severe sepsis in the developed countries has been estimated as being between 28 and 50%, ranging from 15% in patients with sepsis to 40-50% in patients with septic shock with multi-organ dysfunction syndrome (MODS).^9^ It has been documented that sepsis is responsible for 60-80% deaths in developing countries including Pakistan.^10^

Population and the extremes of age’s, infants and children and the elderly are the most vulnerable and so are people with chronic illnesses like diabetes mellitus, kidney and liver disease. The number of cases has been on the rise due to aging population, increasing lifespan of people with chronic illnesses and spread of antibiotic resistant organisms.^11^,^12^

There are a number of studies describing the epidemiology, risk factor and its outcome of severe sepsis and septic shock in various countries worldwide,^7^ in developing countries, unfortunately, data on bacterial sepsis are lacking, especially in adults.^13^ Data on sepsis in Pakistan remains regrettably scanty. To give an example, a nation-wide registry of sepsis does not exist.^14^

In view of the paucity of information concerning sepsis in developing countries, and especially in Pakistan we have developed a study aim to explore the factors associated with outcome among patients with severe sepsis and septic shock admitted to the intensive care unit, Northwest General Hospital and Research Centre Peshawar Pakistan.

## METHOD

This prospective observational study was performed at Medical intensive care unit (MICU) at Northwest General Hospital & Research Centre from February 2014 to October 2015.(21 Months)

### Patient selection

All participants admitted to MICU were screened for eligibility. Patients less than 18 years of age or with MICU length of stay (LOS) less than 24 hours were excluded while those with severe sepsis/septic shock at MICU admission or during hospitalization were included in the study, and only the first episode of severe sepsis or septic shock was counted. Patients readmitted into MICU during the same hospitalization were not screened again. Severe sepsis and septic shock were defined according to the American College of Chest Physicians/Society of Critical Care Medicine consensus conference definitions.^1^ The primary outcome measure was incidence and crude hospital mortality of severe sepsis and septic shock, as well as the risk factors for death. We included patients coming from emergency room and hospital wards covering both community and hospital-acquired infections.

The data was recorded using a predesigned structured format for all new adult admission to ICU. Demographic data, source of infection, comorbid diseases, and clinical laboratory data, hospital stay and outcomes were collected prospectively. Patients were followed up until death or hospital discharge, whichever occurred earlier. Ethical approval for the study was obtained from hospital ethical committee. Data collection was standardized and performed by the main investigator along with trained sub-investigators during the study period.

### Statistical analysis

Data was analyzed using the SPSS 20 software program. Numerical variables were presented as mean±SD. Categorical variables were described as frequency percentage. Binary logistic regression and multi-variate analysis was carried out to identify the factors associated with the survival and mortality of the patients. The level of statistical significance was set at p < 0.05.

## RESULTS

A total of 450 patients admitted to the Medical ICU during the study period were assessed for inclusion in the study sample. Of whom n= 268 patients were diagnosed with sepsis. Majority 147 (54.9%) were male patients with a mean age of 54.8. The presence of septic shock was prevalent in 59.3% with average ICU stay was 5.34 days details are shown in Table-I.

**Table-I:** Baseline/Demographic characteristics of patients.

Age (years) median 58 years	54.85±19.85
*Gender*	
Male	147(54.9%)
Female	121(45.1%)
Length of hospital stay (days)	5.34± 4.23
*Diagnosis*	
Severe sepsis	109(40.7%)
Septic shock	159(59.3%)
Outcome**	
Dead	109(40.7%)
Discharged	159(59.3%)
*Blood culture*	
Positive	91(34%)
Negative	177(66%)
*Urine culture*	
Positive	68(25.4%)
Negative	199(74.3%)
*Sputum culture*	
Positive	49(18.3%)
Negative	193(72%)
Mortality*	
Severe sepsis	8(7.3%)
Septic shock	101(63.5%)

* Calculated with reference to outcome

** therefore the sum will not be 100%

Assessing the situation based on micro-organism isolates from cultures, Escherichia coli ESBL was the most commonly observed bacteria followed by Candida, Acinetobacterbaumannii and Methicillin-resistant Staphylococcus aureus. Overall, the presence of microbes were more frequent among blood samples. (Table-II)

**Table-II:** Micro-organism isolates from cultures.

*Organism*	*Blood*	*Urine*	*Sputum*	*Body fluids*	*Pus*	*Total*
Escherichia coli Extended spectrumbeta lactamase (ESBL)	18	26	6	1	5	56
Acinetobacter baumannii	12	1	11	0	1	25
Methicillin-resistant Staphylococcus aureus	6	0	7	0	5	18
Candida	5	26	1	0	0	31
Methicillin-sensitive Staphylococcus aureus	5	0	1	1	2	9
Providencia species	4	2	0	0	0	6
Enterobacter species	1	5	2	0	0	8
Klebsiella ESBL	0	1	2	0	0	3
Others**	40	7	19	3	0	69

** Mucor, Acid-fast bacilli (AFB), Moraxella, streph, pseudo MDR, Diphtheroid,

Crimean-Congo haemorrhagic fever (CCHF), Enterococcus, Serratia, Bacteroids, E coli, Pseudomons.

The most common source of sepsis was lung infections (42.2%) followed by urinary tract infections (18.7%), soft tissue infections (6.3%) abdominal infections (6%) and in 6.3% patients the source remained unknown. Details are shown in Table-III.

**Table-III:** Site of infection and organ involvements.

*Source*	*Outcome*		*Total*

	*Dead*	*Discharge*	
Lung	51	71	122
Pneumonia with empyema	2	1	3
Urinary tract	10	40	50
CNS	5	9	14
Skin/soft tissue	5	10	15
Abscess	4	3	7
Abdomen	9	7	16
Lung + Urinary tract	8	4	12
Lung + CNS	2	1	2
Unknown	11	6	17
Endocarditis	0	1	1
CCHF	2	0	2
Line sepsis	0	2	2
Genital tract	0	3	3
CNS + Urinary tract	0	1	1

Total	109(40.7%)	159 (59.3%)	267

### Factors associated with the outcome of sepsis management

Using outcome as the dependent variable, binary logistic regression was applied. All the variables commonly reported in literature were added in the model and odd ratios were estimated for potential association with the outcome of the sepsis patients. Results have shown that, gender age, length of hospital stay, was not significantly associated with the outcome of therapy (i.e. death or discharged). However, increase in number of days of hospitalization was observed to be slightly associated with the outcome of the treatment (1.086 [1.002 – 1.178], 0.046). Along with the length of hospital stay, having a positive blood culture was observed to be an important factor associated with the outcome i.e. death / discharge of the patients. Finally the risk of mortality was higher among the patients with septic shock 22.161[10.055 – 48.840], and having respiratory, kidney and central nervous system complications. Details are shown in Table-IV.

**Table-IV:** Factors associated with the sepsis outcome.

*Variables*	*Exp (B)*	*CI[95%]*
Gender	1.079	[0.661 – 1.761]
Age	0.986	[0.975 – 1.007]
Length of hospital stay	0.979	[0.970 – 1.177]
Blood Culture Positive	1.727	[1.034 – 2.884]
Urine Culture Positive	0.351	[ 0.188 – 0.656]
Sputum Culture Positive	0.803	[ 0.420 – 1.534]
Hospital Acquired Infections	0.989	[0.762 – 1.284]
Co-morbidities	1.007	[0.985 – 1.030]
Renal complications/infections during sepsis	4.653	[2.336 – 9.266]
Respiratory complications/infections during sepsis	22.400	[2.831 – 57.266]
CNS complications/infection during sepsis	2.589	[1.357 – 4.939]
Gastro-Intestinal complications/infection during sepsis	2.021	[ 0.720 – 5.673]
Liver complications during sepsis	1.164	[0.483 – 2.803]
Septic shock	22.161	[10.055 – 48.840]

*significant, binary logistic regression was applied.

Dependent variable “outcome”. Model was capable to predict 82.4% of the categories

Furthermore variables found significantly associated with sepsis outcome were added in a linear regression model to predict the effect of change. Overall it is seen that septic shock alone was found responsible for death among 32.0% of the patients (Model 1: R^2^ 0.32, p=0.000). In the final model (model 6), urine culture, kidney complications, CNS infections, Length of Hospital stay in days and respiratory infections were also added to the final model and it was seen that (Model 6: R^2^ 0.421, p=0.016), the risk of mortality further increase by 8.0%. Overall, the factors and the predictors added in model 6 were found to increase the risk of mortality among the patients by 40%. Details are shown in Table-V.

**Table-V:** Predictors of Mortality.

*Model*	*R Square*	*Change Statistics*				

		*F Change*	*Standard Error*	*df2*	*df1*	*p-value*
1	.320	124.810	.407	265	1	.000
2	.354	13.783	.397	264	1	.000
3	.384	12.687	.389	263	1	.000
4	.398	6.381	.385	262	1	.012
5	.408	4.411	.382	261	1	.037
6	.421	5.854	.379	260	1	.016

Model 1: Predictors: (Constant), septic shock;

Model 2: Predictors: (Constant), septic shock, Urine culture

Model 3: Predictors: (Constant), septic shock, Urine culture, kidney complications;

Model 4: Predictors: (Constant), septic shock, Urine culture, kidney complications, CNS infections

Model 5: Predictors: (Constant), septic shock, Urine culture, kidney complications, CNS infections, Length of Hospital in days

Model 6: Predictors: (Constant), septic shock, Urine culture, kidney complications, CNS infections, Length of Hospital in days, respiratory infections

Table-Ι: Patients’ demographics.

## DISCUSSION

The current study is perhaps one of the very few prospective studies aiming to investigate the outcome of sepsis in private health care setting in Pakistan. Of the entire study population about 159 (59.3%) were diagnosed with sepsis shock and 109(40.7%) were found to be suffering through sepsis. It was surprising to see that the mortality rate reported among the severe sepsis patients was 8(7.3%), and remaining 101(92.7%) were treated successfully and discharged. The survival rate among the severe sepsis patient revealed by this study is far higher than the studies done in global ICU setting reporting the mortality rate to be 20-50%.^15^,^16^

Comparing our results with Albert and colleagues (2003) where mortality with severe sepsis was shown to be 40.9%, interestingly our mortality with severe sepsis was low at 7.3%. This could be difficult to explain, however methodological variations in studies may make it very difficult to compare results across. Even applying the same diagnostic criteria but different design of study, inclusion and exclusion criteria may probably account for some of the differences.^17^ However, the mortality rate among the septic shock patients was reported to be very high 101 (63.5%), and only 58(36.5%) patient survived the impact of the septic shock.

One of the recent multicenter study has revealed a cumulative rate of 28.4% among the patient population suffering from sepsis and septic shock.^18^ Comparing our results with this multicenter study it was revealed that the cumulative mortality rate of severe sepsis and septic shock was 40.7%. This is much higher in comparison to the studies done in the developed nations.^19^-^22^ Comparison with Pakistani ICU practice, it is seen that on an average the mortality rate is found to be higher that 30.0%.^4^ However, mortality with septic shock was comparable with (60.5% Albert & Colleagues vs. 63.5% in our study). Thus to compare our results with those of other studies around the globe, it is necessary to take into account all the differences in our study population. Quality Primary care here is patchy and early recognition of severity of illness is often missed. Our patients may present very late for a number of reasons including financial restrains and travelling from far away countryside. However, this situation somehow is in line with some observational studies^23^,^24^ that suggest the mortality rate may still be higher than reported from interventional studies^25^ that often exclude the highest risk groups of patients, and also more formally structure the delivery of care.

### Causes of Mortality

This is perhaps one of the challenging task to identify the factors associated with the mortality of patients. Variety of factors may have association with the survival and death of patients suffering from sepsis and septic shock. Addressing the results of the current study, it was seen that about 54.9% of the patients were male. However, being a female was observed to be slightly contributing to mortality 1.070 [0.655 – 1.749]. In our study, we observed adult patients with sepsis for the entire duration of hospitalization. Our patients were younger, with a median of 58 years as compared with sepsis patients of European origin [median 64 years]^5^ those from North America [mean 60.8 years]^26^ And Brazil [median age 65.2 years].^27^ Thus the importance of clinical parameters will be perhaps more significant to the mortality rather than a gender comparison. It was noticed that in about 34.0% of the cases blood culture was positive for microbial growth, and in 25.4% cases urine culture was positive. While sputum culture was positive in about 18.3% of cases. Having a blood culture positive was more likely to be associated with the mortality 1.964 [1.101 – 3.503]. Moreover, the chance of mortality was at least 35% higher among the patients with positive urine culture 0.351 [0.188 – 0.656]. of whom 50(19.3%) were with E.coli positive culture. These results confirm the results of the one of the recent study that reported higher mortality among the patients with UTIs and E. Coli positive culture.^4^

Overall analysis revealed that odds of surviving for an elderly patients is less 0.986 CI [0.975 – 1.007]. However, chance of survival reduces by 0.979 percent with an increase in length in hospital stay. Estimating the impact of septic shock on survival of patients, it was noticed that the likelihood for death was 22.161[10.055 – 48.840] times higher among the patients suffering from septic shock, similarity the chance of death further increase by 22.400 [2.831 – 57.266] if patient have respiratory complications or lungs involvement CNS and Urinary tract infections alone or in conjunction with one another. Thus chance of mortality was highest when patient have septic shock and respiratory and urinary tract infection.^4^

Finally, multivariate modeling revealed that septic shock alone may result in an increase in mortality by 32.0% (R^2^ 0.320, Model 1). in addition the chance of survival further decrease by 10.0% when patient have positive urine culture, kidney complications, CNS and respiratory infections and longer stay in hospital (Model 6). According to the literature, the most common infection sites in septic patients are the respiratory and genitourinary systems as well intra-abdominal surgical infections and indwelling catheters.^15^ Our results show similar findings; the most common focus of infection was Respiratory (pneumonia in 42.2%) followed by urinary tract (18.7%), soft tissue infections (6.3%) abdomen (6%) and in 6.3% patients the source remained unknown. The mortality was significantly higher in patients with pneumonia (40.7%). with Gram-negative organisms being the most predominant culprits.

## CONCLUSION

Sepsis constitutes the majority of the patients admitted to the medical intensive care unit and carries a significant mortality. We have shown a prospective record of patients with severe sepsis and septic shock who were admitted to medical intensive care unit of our hospital. Results of this study revealed that the chances of recovery were poor among the patients with septic shock with a higher risk of mortality. Pneumonia and urinary tract infections were the predominant causes.

## References

[1] Reinhart K, Daniels R, Machado FR (2013). The burden of sepsis:a call to action in support of World Sepsis Day 2013. Rev Bras de terintensiva.

[2] Vincent JL (2012). Increasing awareness of sepsis:World sepsis day. Crit Care.

[3] Ortíz G, Dueñas C, Rodríguez F, Barrera L, de La Rosa G, Dennis R (2014). Epidemiology of sepsis in Colombian intensive care units. Biomédica.

[4] Hashmat N, Shabbir I, Rahat T, Ijaz F, Majeed S (2015). Clinical Profile and Disease Outcome of Septic Patients at Public Sector Hospital. Pak J Med Res.

[5] Vincent J-L, Sakr Y, Sprung CL, Ranieri VM, Reinhart K, Gerlach H (2006). Sepsis in European intensive care units:Results of the SOAP study. Crit Care Med.

[6] Carrigan SD, Scott G, Tabrizian M (2004). Toward resolving the challenges of sepsis diagnosis. Clin Chem.

[7] Zhou J, Qian C, Zhao M, Yu X, Kang Y, Ma X (2014). Epidemiology and outcome of severe sepsis and septic shock in intensive care units in mainland China. PLoS ONE.

[8] Quartin AA, Calonge RO, Schein RM, Crandall LA (2008). Influence of critical illness on physicians’prognoses for underlying disease:A randomized study using simulated cases. Crit Care Med.

[9] Martin GS (2012). Sepsis, severe sepsis and septic shock:changes in incidence, pathogens and outcomes. Expert Rev Anti Infect Ther.

[10] (2014). Sepsis Fact in world sepsis day.

[11] Levinson AT, Casserly BP, Levy MM (2011). Reducing mortality in severe sepsis and septic shock. Semin Respir Crit Care Med.

[12] Sepsis Fact Sheet (2014). National Institute of General Medicine January.

[13] Cheng AC, West TE, Limmathurotsakul D, Peacock SJ (2008). Strategies to reduce mortality from bacterial sepsis in adults in developing countries. PLoS Med.

[14] Siddiqui S, Jamil B, Nasir N, Talat N, Khan FA, Frossard P (2013). Characteristics and outcome of sepsis–A perspective from a tertiary care hospital in Pakistan. IJSER.

[15] Angus DC, Linde-Zwirble WT, Lidicker J, Clermont G, Carcillo J, Pinsky MR (2001). Epidemiology of severe sepsis in the United States:Analysis of incidence, outcome, and associated costs of care. Crit Care Med.

[16] Dombrovskiy VY, Martin AA, Sunderram J, Paz HL (2007). Rapid increase in hospitalization and mortality rates for severe sepsis in the United States:A trend analysis from 1993 to 2003. Crit Care Med.

[17] Albert C, Brun-Buisson C, Goodman SV, Guidici D, Granton J, Moreno R (2003). Influence of systemic inflammatory response syndrome and sepsis on out- come of critically Ill infected patients. Am J Respir Crit Care Med.

[18] Rhodes A, Phillips G, Beale R, Cecconi M, Chiche JD, De Backer D (2015). The Surviving Sepsis Campaign bundles and outcome:results from the International Multicentre Prevalence Study on Sepsis (the IMPreSS study). Intensive Care Med.

[19] Kaukonen KM, Bailey M, Suzuki S, Pilcher D, Bellomo R (2014). Mortality related to severe sepsis and septic shock among critically ill patients in Australia and New Zealand 2000-2012. JAMA.

[20] Stevenson EK, Rubenstein AR, Radin GT, Wiener RS, Walkey AJ (2014). Two decades of mortality trends among patients with severe sepsis:a comparative meta-analysis. Crit Care Med.

[21] Peake SL, Delaney A, Bailey M, Bellomo R, Cameron PA, Cooper DJ (2014). Goal directed resuscitation for patients with early septic shock. N Eng J Med.

[22] Yealy DM, Kellum JA, Huang DT, Barnato AE, Weissfeld LA, Pike F (2014). A randomized trial of protocol-based care for early septic shock. N Eng J Med.

[23] Phua J, Koh Y, Du B, Tang YQ, Divatia JV, Tan CC (2011). Management of severe sepsis in patients admitted to Asian intensive care units:prospective cohort study. BMJ.

[24] Levy MM, Rhodes A, Phillips GS, Townsend SR, Schorr CA, Beale R (2014). Surviving Sepsis Campaign:association between performance metrics and outcomes in a 7.5-year study. Intensive Care Med.

[25] Ranieri VM, Thompson BT, Barie PS, Dhainaut JF, Douglas IS, Finfer S (2012). Drotrecoginalfa (activated) in adults with septic shock. N Eng J Med.

[26] Khan NA, Palepu A, Norena M, Ayas N, Wong H, Chittock D (2008). Differences in hospital mortality among critically ill patients of Asian, Native Indian, and European descent. Chest J.

[27] Záhorec R, Firment J, Straková J, Mikula J, Malík P, Novák I (2005). Epidemiology of severe sepsis in intensive care units in the Slovak Republic. Infection.

